# Clinically relevant enhancement of human sperm motility using compounds with reported phosphodiesterase inhibitor activity

**DOI:** 10.1093/humrep/deu196

**Published:** 2014-08-14

**Authors:** Steve Tardif, Oladipo A. Madamidola, Sean G. Brown, Lorna Frame, Linda Lefièvre, Paul G. Wyatt, Christopher L.R. Barratt, Sarah J. Martins Da Silva

**Affiliations:** 1 Reproductive and Developmental Biology, Medical School, Ninewells Hospital, University of Dundee, DundeeDD1 9SY, UK; 2 Assisted Conception Unit, NHS Tayside, Ninewells Hospital, Dundee DD1 9SY, UK; 3 University of Abertay, Dundee DD11HG, UK; 4 Medical School, University of Birmingham, Birmingham B152TG, UK; 5 Drug Discovery Unit, College of Life Sciences, University of Dundee, Dundee, UK; 6 Present address: International Center for Biotechnology, MOFA Global, Mount Horeb, WI 53572, USA

**Keywords:** sperm, male fertility, phosphodiesterase inhibitors, sperm motility, drug discovery

## Abstract

**STUDY QUESTION:**

Can we identify compound(s) with reported phosphodiesterase inhibitor (PDEI) activity that could be added to human spermatozoa *in vitro* to enhance their motility without compromising other sperm functions?

**SUMMARY ANSWER:**

We have identified several compounds that produce robust and effective stimulation of sperm motility and, importantly, have a positive response on patient samples.

**WHAT IS KNOWN ALREADY:**

For >20 years, the use of non-selective PDEIs, such as pentoxifylline, has been known to influence the motility of human spermatozoa; however, conflicting results have been obtained. It is now clear that human sperm express several different phosphodiesterases and these are compartmentalized at different regions of the cells. By using type-specific PDEIs, differential modulation of sperm motility may be achieved without adversely affecting other functions such as the acrosome reaction (AR).

**STUDY DESIGN, SIZE, DURATION:**

This was a basic medical research study examining sperm samples from normozoospermic donors and subfertile patients attending the Assisted Conception Unit (ACU), Ninewells Hospital Dundee for diagnostic semen analysis, IVF and ICSI. Phase 1 screened 43 commercially available compounds with reported PDEI activity to identify lead compounds that stimulate sperm motility. Samples were exposed (20 min) to three concentrations (1, 10 and 100 µM) of compound, and selected candidates (*n* = 6) progressed to Phase 2, which provided a more comprehensive assessment using a battery of *in vitro* sperm function tests.

**PARTICIPANTS/MATERIALS, SETTING, METHODS:**

All healthy donors and subfertile patients were recruited at the Medical Research Institute, University of Dundee and ACU, Ninewells Hospital Dundee (ethical approval 08/S1402/6). In Phase 1, poor motility cells recovered from the 40% interface of the discontinuous density gradient were used as surrogates for patient samples. Pooled samples from three to four different donors were utilized in order to reduce variability and increase the number of cells available for simultaneous examination of multiple compounds. During Phase 2 testing, semen samples from 23 patients attending for either routine diagnostic andrology assessment or IVF/ICSI were prepared and exposed to selected compounds. Additionally, 48 aliquots of prepared samples, surplus to clinical use, were examined from IVF (*n* = 32) and ICSI (*n* = 16) patients to further determine the effects of selected compounds under clinical conditions of treatment. Effects of compounds on sperm motility were assessed by computer-assisted sperm analysis. A modified Kremer test using methyl cellulose was used to assess sperm functional ability to penetrate into viscous media. Sperm acrosome integrity and induction of apoptosis were assessed using the acrosomal content marker PSA-FITC and annexin V kit, respectively.

**MAIN RESULTS AND THE ROLE OF CHANCE:**

In Phase 1, six compounds were found to have a strong effect on poor motility samples with a magnitude of response of ≥60% increase in percentage total motility. Under capacitating and non-capacitating conditions, these compounds significantly (*P* ≤ 0.05) increased the percentage of total and progressive motility. Furthermore, these compounds enhanced penetration into a cervical mucus substitute (*P* ≤ 0.05). Finally, the AR was not significantly induced and these compounds did not significantly increase the externalization of phosphatidylserine (*P* = 0.6, respectively). In general, the six compounds maintained the stimulation of motility over long periods of time (180 min) and their effects were still observed after their removal. In examinations of clinical samples, there was a general observation of a more significant stimulation of sperm motility in samples with lower baseline motility. In ICSI samples, compounds #26, #37 and #38 were the most effective at significantly increasing total motility (88, 81 and 79% of samples, respectively) and progressive motility (94, 93 and 81% of samples, respectively). In conclusion, using a two-phased drug discovery screening approach including the examination of clinical samples, 3/43 compounds were identified as promising candidates for further study.

**LIMITATIONS, REASONS FOR CAUTION:**

This is an *in vitro* study and caution must be taken when extrapolating the results. Data for patients were from one assessment and thus the robustness of responses needs to be established. The *n* values for ICSI samples were relatively small.

**WIDER IMPLICATIONS OF THE FINDINGS:**

We have systematically screened and identified several compounds that have robust and effective stimulation (i.e. functional significance with longevity and no toxicity) of total and progressive motility under clinical conditions of treatment. These compounds could be clinical candidates with possibilities in terms of assisted reproductive technology options for current or future patients affected by asthenozoospermia or oligoasthenozoospermia.

**STUDY FUNDING/COMPETING INTEREST(S):**

This study was funded primarily by the MRC (DPFS) but with additional funding from the Wellcome Trust, Tenovus (Scotland), University of Dundee, NHS Tayside and Scottish Enterprise. The authors have no competing interests. A patent (#WO2013054111A1) has been published containing some of the information presented in this manuscript.

## Introduction

Sperm dysfunction has long been acknowledged as the single most common cause of infertility (Hull *et al.*, 1985; Irvine, 1998), yet there is currently no drug a man can take to significantly improve his fertility. The only option is assisted reproductive technology (ART) which usually consists of a graduation of treatment depending on severity, i.e. intrauterine insemination (IUI) for mild, *in vitro* fertilization (IVF) for moderate and intra-cytoplasmic sperm injection (ICSI) for men with severe, sperm dysfunction (Barratt *et al.*, 2011). A primary manifestation of sperm dysfunction is poor motility which negatively impacts on successful ART (Publicover and Barratt, 2011; Van der Steeg *et al.*, 2011; Tomlinson *et al.*, 2013). Before ICSI, the various strategies to improve the IVF fertilization rate by chemical stimulation of spermatozoa primarily involved treatment with non-specific inhibitors of phosphodiesterases (PDEIs, e.g. pentoxifylline (PTX)), a family of related phosphohydrolases that selectively catalyse hydrolysis of the 3′ cyclic phosphate bonds of cAMP or cGMP. A landmark study demonstrated a significant enhancement of fertilization rates when using PTX (Yovich *et al.*, 1990) and 77 pregnancies were achieved (Yovich, 1993). Two IUI studies later reported higher pregnancy rates following sperm stimulation with PTX combined with IUI but the data were not part of a controlled study (Negri *et al.*, 1996; Stone *et al.*, 1999). However, the results using PTX have been inconsistent. For example, Tournaye *et al.* showed a lower fertilization rate *in vitro* following PTX treatment, probably due to the nature of the patients selected (moderate male factor infertility versus previous unsuccessful IVF treatment) and/or the protocols of PTX addition (Tournaye *et al.*, 1994). Following development of ICSI, which has now become the treatment of choice for severe male factor infertility, the concept of sperm stimulation for IVF/IUI has fallen from grace.

Previously published *in vitro* studies using PTX provide explanations concerning its potential limitations. For example, Tesarik *et al.* examined the use of PTX (final concentration 1 mg/ml) on *in vitro* sperm motility in 14 normal men and 25 men with asthenozoospermia. Consistent with other authors, PTX did not affect the percentage of motile cells but significantly increased velocity. This occurred in all the men with normal semen analysis and in the majority of men (21/25) with asthenozoospermia. In the latter, all men showed an increase in hyperactivation. However, in studies using PTX, a significant disadvantage has been the premature stimulation of the acrosome reaction (AR) (Tesarik *et al.*, 1992; Yovich, 1993; Ford *et al.*, 1994). This has discouraged further studies on the modulation of cAMP for clinical applications.

It is now clear that there are multiple forms of phosphodiesterases (PDEs) with different kinetic and regulatory properties, classified into 11 different families and comprising 21 different gene products. Indeed, there are estimated to be well over 100 mRNA products, as well as multiple proteins transcribed from these genes, due to alternative transcription start sites and splicing of precursor molecules (Francis *et al.*, 2011). Spermatozoa contain several different PDEs, and the regulation of intracellular cAMP and cGMP is therefore largely specified by the exact nature and localization of the expressed PDEs (Lefièvre *et al.*, 2000; Lefièvre *et al.*, 2002). Surprisingly, in view of the plethora of existing and new-generation PDEIs (Francis *et al.*, 2011), there are very few studies examining the inhibition of specific PDEs in human sperm. Fisch *et al.*, (1998) examined the biological activity of PDE-1 and PDE-4 in the sperm of 30 subfertile men. PDE-4 inhibition (via Rolipram) selectively increased the percentage of motile cells at 2 and 24 h of incubation, with the most significant effects noted in those samples with the lowest concentration of motile cells. PDE-1 inhibitors (for example, 8-MeO-IBMX) selectively activated AR, but this was not the case with inhibition of PDE-4. The data on the role of PDE-5 are controversial, probably because PDE-5 represents only a very small fraction of the PDE activity (Lefièvre *et al.*, 2000). Conflicting biological effects have been observed using PDE-5-specific inhibitors, sildenafil and tadalafil, *in vivo* (Sousa *et al.*, 2014; Yang *et al.*, 2014). Induction of the AR (presumably due to an increase, above a critical threshold, of cGMP) is frequently reported (Glenn *et al.*, 2007).

In an attempt to discover novel therapeutics for male factor subfertility, the first objective of an effective drug discovery programme would be to identify (or develop) key compounds that stimulate sperm motility without compromising their fertilizing capacity, e.g. by causing premature stimulation of AR. The clinical objective was to identify compounds that increase the number of functional sperm in the vicinity of the oocyte, based on the hypothesis that this increase would lead to a higher chance of fertilization, thus achieving an increase in live birth rate (see Publicover and Barratt, 2011). If achieved, this would also increase the availability of fertility treatments to a global population, i.e. by using IUI instead of IVF and by making cost-effective IUI available to a much wider patient population. There is significant data to support this concept. A large number of studies demonstrate a significant relationship between conception *in vivo* and the number/concentration of motile sperm and/or percentage motile cells in subfertile couples (reviewed in Tomlinson *et al.*, 1999, 2013). Indeed, the same applies in donor insemination. Perhaps, the most comprehensive data are from CECOS (Centres d’étude et de conservation des œufs et du sperme humains) that demonstrates an almost doubling of success rates (∼13 versus ∼7%) per cycle of treatment when the number of motile sperm in the straws for insemination was 5–10 million rather than <5 million (David *et al.*, 1980). For IUI, there is a significant relationship between conception and number/concentration of motile sperm and/or the percentage of motile cells in the semen used for insemination. For example, Horvath *et al.* presented a linear relationship between total motile sperm in post-preparation samples and conception rates (Horvath *et al.*, 1989). Experiments in natural conception, IUI and IVF also consistently demonstrate that the most significant clinical relationship is observed at the lower ends of the spectrum, e.g. with lower numbers of motile cells/low progression (Publicover and Barratt, 2011). For example, Hargreave *et al.*, when assessing natural conception, suggested that a doubling of the motile sperm concentration from 2 to 4 million/ml results in a 2.5-fold increase in pregnancy rates (adapted from Comhaire, 2000; Publicover and Barratt, 2011), yet at higher sperm concentrations the positive effect was relatively small. Importantly for this strategy, by increasing the number of motile cells in the vicinity of the oocyte significantly increased IVF success and reduced the incidence of fertilization defects in men with sperm dysfunction (Tournaye *et al.*, 2002). Thus, in the potential target clinical population (men with sperm generally below WHO 2010 (Cooper *et al.*, 2010) thresholds of motility), there would only need to be a relatively moderate increase in motility to potentially have a noticeable clinical benefit. If successful, this strategy would simplify and increase access to treatment (Lefièvre *et al.*, 2007). Increasing success rates of IUI and/or IVF would reduce dependence on ICSI and have a substantial cost saving. For example, converting ∼10% of the ∼61 000 IVF/ICSI cycles done annually in the UK (HFEA, 2013) to intervention-supported IUI would save up to £27.5 million per annum, with proportionate savings worldwide.

The objective of this study was to systematically and comprehensively screen a series of 43 commercially available compounds with reported PDEI activity to identify key candidates that could be added to human spermatozoa *in vitro* to enhance their motility. Using a two-stage approach, we have successfully identified several compounds that have robust and effective stimulation of sperm motility (i.e. functional significance with longevity and no toxicity) and, importantly, have a positive response on patient samples. These compounds could be subjected to further study for potential clinical use and/or chemically modified to improve efficacy. The experiments presented provide an exciting first step towards the clinical goal of robust and effective *in vitro* sperm cell stimulation.

## Materials and Methods

### Overall experimental design

**Figure 1:**
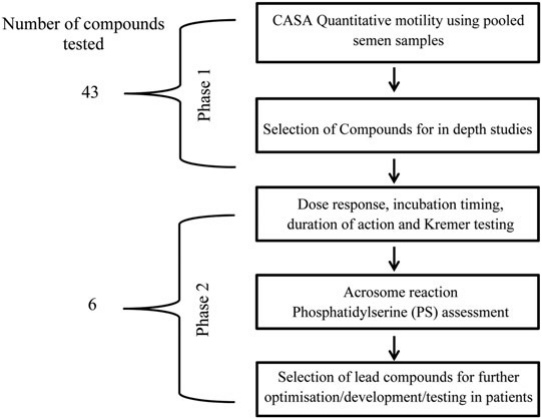
Schematic diagram of overall experimental design: 43 compounds were identified and selected at the DDU, University of Dundee. Effects of compounds on the kinematic parameters of human spermatozoa were assessed using CASA. Compounds that have robust and effective stimulation on sperm motility were selected for further testing (Phase 2) to determine their effect(s) on sperm function, including experiments on patient samples.

**Figure 2:**
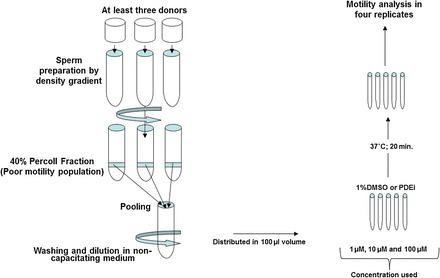
Procedure performed to screen different compounds in Phase 1: samples from three to four donors were used to isolate the 40% fraction (poor motility population) by density gradient centrifugation. Following sperm preparation, cells were pooled together and 100 µl was used to assess quantitative sperm motility following 20 min incubation with 100 µM final concentration of compound(s) in NCM.

We used a two-phased approach (Fig. 1). Phase 1 screened 43 commercially available compounds with reported PDEI activity in order to identify lead compounds that stimulate sperm motility (Fig. 2). These lead compounds (*n* = 6) progressed to Phase 2 which provided a comprehensive assessment using a series of *in vitro* sperm function tests designed to determine if the compounds could be of potential clincial value (see Mortimer *et al.*, 2013). All screening was performed blind. The codes to the compounds are presented in Supplementary data, Table SI.

### Ethical approval

Written consent was obtained from each patient in accordance with the Human Fertilization and Embryology Authority (HFEA) Code of Practice (version 8) under local ethical approval (08/S1402/6) from the Tayside Committee of Medical Research Ethics B. Similarly, volunteer sperm donors (healthy men randomly selected from the general public with no known fertility problems) were recruited in accordance with the HFEA Code of Practice (version 8) under the same ethical approval.

### Study subjects

Semen samples were obtained from samples from healthy research donors (with a normal sperm concentration and motility according to WHO 2010 criteria). These samples were used in Phase 1 and Phase 2 of the study. Patient samples used in Phase 2 were from subfertile patients who underwent diagnostic investigations and/or treatment at the Assisted Conception Unit (ACU), Ninewells Hospital, Dundee, Scotland.

### Semen samples

Semen samples from donors and patients were collected by masturbation into a sterile plastic container after 2–3 days of sexual abstinence. The samples were used for analysis after liquefaction of the semen at 37°C for ∼30 min and within 1 h of production. Semen samples obtained from patients were assessed for the semen profile by clinical embryologists and selected for IVF or ICSI according to clinical indications and semen quality. For the latter, although not always, men with ∼1 × 10^6^ progressively motile cells post-preparation were allocated to IVF and those below this limit were allocated to ICSI. The surplus of the clinical sample used in the IVF or ICSI treatment process was used for analysis (Alasmari *et al.*, 2013b).

### Preparation and analysis of patient samples

In the ACU, commercially available media were used for sperm preparation. The spermatozoa were separated from semen by density gradient centrifugation (40%:80%) using PureSperm™ (Nidacon, Mölndal, Sweden) diluted with Quinn's Advantage Medium with HEPES (SAGE In-Vitro Fertilization; Pasadena, CA, USA). After centrifugation, the pellet was washed by centrifugation at 500*g* for 10 min in 4 ml of Quinn's Advantage Medium with HEPES. If the samples were assigned for IVF, following centrifugation, the supernatant was discarded and pellet was resuspended in Quinn's Advantage Fertilization medium. If the sample was allocated for ICSI, the cells were washed and prepared in Quinn's Advantage Medium with HEPES (Alasmari *et al.*, 2013a,b).

### Chemicals

Compound(s) were purchased from Tocris Bioscience (Bristol, UK), Sigma-Aldrich (Dorset, UK) or Santa Cruz Biotechnology (Heidelberg, Germany) by the Drug Discovery Unit (DDU) at the College of Life Sciences (University of Dundee, UK). The compounds were coded (#1–43 by DDU) and tested for sperm function with an initial primary focus on sperm motility. The codes were broken once all experiments were completed. In Phase 1, 43 compounds were analysed in three independent experiments using 1, 10 and 100 µM final concentrations of compounds. Only the results for the 100 µM experiments are reported.

### Media and donor sperm preparation

All chemicals were purchased from Sigma-Aldrich. Two different media were used (i) not supporting sperm capacitation [non-capacitating media (NCM)]: 1.8 mM CaCl_2_, 5.4 mM KCl, 0.8 mM MgSO_4_.7H_2_O, 116.3 mM NaCl, 1.0 mM NaH_2_PO_4_, 5.55 mM d-glucose, 2.73 mM sodium pyruvate, 41.75 mM sodium lactate, 25 mM HEPES and 3 mg/ml BSA; (ii) supporting capacitation [capacitating media (CM)]: 1.8 mM CaCl_2_, 5.4 mM KCl, 0.8 mM MgSO_4_.7H_2_O, 116.3 mM NaCl, 1.0 mM NaH_2_PO_4_, 5.55 mM d-glucose, 2.73 mM sodium pyruvate, 25 mM sodium lactate, 26 mM sodium bicarbonate and 3 mg/ml BSA (see Alasmari *et al.*, 2013a). In Phase 1, semen samples were obtained from healthy donors and two different sperm populations were isolated using a 40–80% discontinuous density gradient procedure. Briefly, 1 ml of semen was loaded after 30 min of liquefaction at 37°C on the top of a colloidal silica suspension (PureSperm™) made of 80 and 40% layered (2 ml each). The density gradient was centrifuged at 300*g* for 20 min. High-quality cells were in the 80% fraction and the poor motility population was recovered at the interface of 40–80%, respectively, called the 80 and 40% fractions. Both fractions were washed in NCM by centrifugation for 5 min at 500*g* and the sperm pellet was re-suspended using NCM or CM at ∼20 × 10^6^ cells/ml. Sperm cells from three to five different donors were pooled together after sperm preparation in order to obtain enough cells to screen four to five compounds at the same time, thus reducing variability. The first screening was performed with NCM as these are the conditions commonly used in clinics for IUI (Bjorndahl *et al.*, 2010). In Phase 1, the experiments were mainly performed on the 40% fractions (sperm cells with poor motility), which were used as putative surrogates for patient samples. Previous studies have suggested that sperm cells from this fraction have a similar profile in terms of motility, morphology and DNA status to men with male infertility (O'Connell *et al.*, 2003; Glenn *et al.*, 2007).

### Motility assessment and compound(s) treatment

Once the spermatozoa were isolated, they were mixed with DMSO (vehicle; 1% final concentration) or with 1, 10 or 100 µM final concentrations of compound(s). Sperm cells were incubated for various times at 37°C (for cells in NCM) or in a 5% CO_2_ humidified atmosphere (for cells in CM) and the motility was evaluated using a computer-assisted-sperm analysis (CASA) [CEROS machine (version 12), Hamilton Thorne Research, Beverly, MA, USA] attached to an external microscope. Sperm motion characteristics were assessed under a negative phase contrast objective as previously described (Alasmari *et al.*, 2013a). System parameter settings for these analyses were 30 frames at 60 frames per second (Hz); minimum contrast 80; minimum size 3 (pix); upper and lower gates of 0.39 and 1.4 for intensity; and 0.85 and 4.24 for size and the default values for non-motile cells were 6 and 160 for size and intensity, respectively. A minimum of 16 data points were used for tracking a cell. Spermatozoa with an average path velocity >25 µm/s and 80% straightness are considered progressive, while those with a curvilinear velocity (VCL) >150 µm/s, amplitude of lateral head displacement >7.0 µm and decrease in linearity [(straight line velocity/VCL) × 100] (LIN) <50% are defined as hyperactivated cells. Spermatozoa were examined in four-chamber slides of 20 µM deep (Vitrolife, Sweden). In Phase 1, sperm motility was evaluated using a pool of three four semen samples per treatment. After each treatment, aliquots were taken from each pool and loaded on the chamber slides with ∼200 sperm cells analysed at each section of the four-chamber slides. This was performed on three separate occasions with different pooled samples (Fig. 2). In Phase 2, individual semen samples were used.

### Acrosomal status evaluation

Prepared sperm cells (as for donor sperm above) were incubated in the absence or in the presence of compound (100 µM final concentration) in NCM for up to 5 h of incubation. The sample was divided into two where one was incubated with 10 µM of calcium ionophore A23187 (Sigma) and the other half was incubated with the vehicle (1% DMSO), both for 15 min at 37°C. Once complete, cells were smeared, dried onto microscope slides, fixed and permeabilized with 100% methanol and incubated at room temperature for 30 min. The percentage of acrosome reacted cells was evaluated by using fluorescein–isothiocyanate conjugated *Pisum sativum lectin* (PSA-FITC) as previously described (Bjorndahl *et al.*, 2010). Briefly, smeared spermatozoa were incubated with PSA-FITC (100 µg/ml) in the dark for 20 min. The slides were washed with Tris-buffered saline (10 mM Tris–HCl pH 7.4, 150 mM NaCl), mounted (Hydromount, National Diagnostics) with cover slips and at least 200 cells were scored as ‘acrosome intact’ or ‘acrosome reacted’.

### Phosphatidyl translocation determined by annexin V/propidium iodide

As an indicator of apoptosis, the translocation of PS from the inner to outer leaflet of the sperm plasma membrane was assessed by annexin V staining kit (Martin *et al.*, 2005). Briefly, prepared sperm cells were incubated with or without compound (100 µM) in NCM for 20 min. After 20 min of incubation, cells were incubated with annexin-FITC (50 µg/ml)/propidium iodide (100 µg/ml) in NCM and 20 µl of this sperm solution was mounted on a microscope slide and viewed under UV excitation with FITC filters. A minimum of 200 cells were scored per slide.

### Penetration assay (Kremer test)

A modified Kremer tests using methyl cellulose (4000 cP: Sigma-Aldrich, M0512) was used to assess sperm functional ability to penetrate into viscous media as previously described (Ivic *et al.*, 2002; Alasmari *et al.*, 2013b). Briefly, sperm cells were previously treated with compound as described above and glass tubes filled with methyl cellulose was placed vertically in the sperm solution. Glass tubes (5 cm × 0.8 cm × 2 mm; Vitrocom, Mountain Lakes, NJ, USA) were loaded with methyl cellulose by capillary action and the tubes were incubated with the sperm cells at 37°C for 1 h 15 min. The number of sperm cells was scored at 1 cm. The results are expressed as a penetration index [number of spermatozoa observed with treatment/the number of spermatozoa without treatment (control)].

### Removal of compound after 20 min incubation

The longevity of motility as a result of incubation with the compound was tested following washing of the samples. This process was used to simulate the clinical protocols where the compounds would be washed prior to insemination (e.g. IUI) into the female tract or addition of sperm to the oocytes in IVF. Sperm cells with poor motility were isolated as previously described (see Fig. 2) and incubated with selected compounds for 20 min, then the samples were washed (5 min at 300*g*). Samples were then examined for up to 300 min.

### Statistical analysis

Significance of differences between compound(s) was determined by ANOVA using the statistical package PASW Statistics for Windows (Version 18.0., SPSS, Inc., Chicago, IL, USA). The Fisher protected least significant difference test was conducted when the main effect was significant (*P* < 0.05) to determine which treatments were significantly different. The normal distribution was determined by Kolmogorov–Smirnov test and the homogeneity of variance was tested by using the Levene statistical test. Multiple comparisons were analysed using the Tukey's test. For the analysis of individual patient samples, an individual positive response for either % total or % progressive motility was recorded when the values following compound stimulation were noticeably different from control (basal) levels, i.e. when the 2× standard deviations did not overlap.

## Results

Phase 1 screened 43 commercially available compounds with reported PDEI's activity to identify lead compounds that stimulate sperm motility. The selected candidates (*n* = 6) progressed to Phase 2 which provided a more comprehensive assessment using a battery of *in vitro* sperm function tests.

### Phase 1

**Figure 3:**
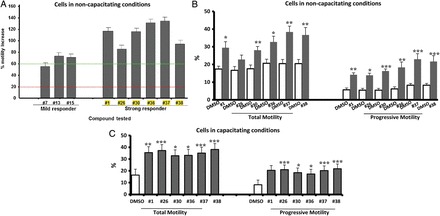
Effect(s) of compound(s) on sperm motility: spermatozoa with poor motility from the 40% fraction were treated for 20 min at 37°C with 100 µM of compound. (**A**) Responses arbitrarily categorized into: strong responder (#1, #26, #30, #36, #37 and #38) and mild responder (#7, #13, #15), where a 100% increase equals a 2-fold increase in motility compared with 1% DMSO negative control (i.e. if DMSO = 15%, treatment = 30%). *n* = 3 (three separate analysis of pooled sample), mean ± SEM (see Supplementary data, Fig. S1a–d for other compounds tested). The green line indicates threshold for strong responder (≥60% increase), while the red line is the threshold for background. *X*-axis title with yellow colour indicates the most promising compounds. (**B**) Effect(s) of selected compounds on percentage of motile and progressively motile cell under non-capacitating conditions (*P ≤ 0.05, **P ≤ 0.001 and ***P ≤ 0.0001 in comparison with control), *n* = 6 (six different samples from six individuals), mean ± SEM. (**C**) Sperm cells were incubated at 37°C in a 5% CO_2_ humidified atmosphere for 3 h; they were then treated with 100 µM of compound and left to incubate for 20 min at 37°C in a 5% CO_2_ humidified atmosphere (*P ≤ 0.05, **P ≤ 0.001 and ***P ≤ 0.0001 in comparison with control), *n* = 7 (seven different samples from seven individuals), mean ± SEM.

The initial screening using the poor motility fraction from pooled donor samples identified a number of compounds with a significant effect on motility (total and progressive motility) (Fig. 3A, Supplementary data, Fig. S1). These were artificially classed as moderate (20–60% increase in total motility) and strong effects (>60% increase in total motility) (Supplementary data, Fig. S1). At the end of Phase 1, six compounds (# 1, #26, #30, #36, #37 and #38) were identified to have a strong effect on total and progressive motility and were chosen for further evaluation in Phase 2 (Fig. 3A).

### Phase 2

#### Individual samples

Examination of the compounds on individual samples showed a significant increase in total and progressive motility for all compounds (except #26 for total motility) (Fig. 3B). There was no significant difference between the compounds regarding the effect on total or progressive motility (Fig. 3B). Similar significant increases in total and progressive motility were observed compared with the pooled samples in Phase 1 (compare Fig. 3A and B).

#### Penetration assay (Kremer test)

**Figure 4:**
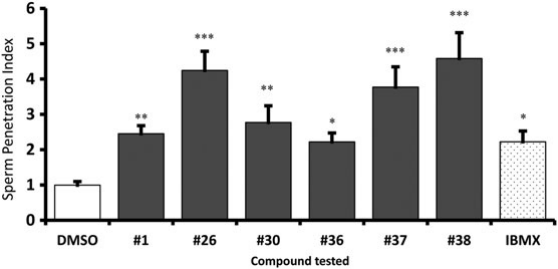
Penetration of spermatozoa treated with compound into viscous media: sperm cells from 40% fraction (poor motility population) were treated with or without 100 µM final concentration of compound(s) [1% DMSO-Control, 500 µM IBMX (3-isobutyl-1-methylxanthine)-positive control]. Treated cells were allowed to penetrate into methyl cellulose solution for 1 h 15 min (Kremer test) (*P ≤ 0.05, **P ≤ 0.001 and ***P ≤ 0.0001 in comparison with control), *n* = 8 (eight different samples from eight individuals), mean ± SEM.

There were significantly higher numbers of cells at 1 cm when sperm were incubated in any of the six compounds compared with vehicle control (Fig. 4). Cells incubated in compounds #26 and #38 showed a significantly higher response than #1 and #36. Cell incubated in compound #38 also showed a significantly higher response than those incubated in #30, whilst those incubated in compound #37 showed no significant differences compared with the other compounds.

#### AR and PS externalization

There was no significant induction of the AR (*P* = 0.6) or of PS externalization (*P* = 0.6) after continuous incubation with any of the compounds compared with vehicle controls (Supplementary data, Fig. S2).

#### Experiments in CM

Sperm cells incubated in capacitating conditions with each of the compounds showed a significant increase in % total and % progressive motility (*n* = 7 Fig. 3C) compared with controls. The increase was comparable to that in cells incubated under non-capacitating conditions (*n* = 6 samples; compare Fig. 3B and C).

#### Longevity of effect following continuous incubation

Phase 1 tested the motility after a 20 min incubation period. When sperm samples were continuously incubated with compounds over a longer period of time (up to 180 min), there was generally a significantly higher total and progressive motility compared with the control (Table I).

**Table I:** Effects of compounds on sperm motility with continuous co-incubation (180 min) under non-capacitation conditions.

	Total motility (% ± SEM)							Progressive motility (% ± SEM)						
	DMSO	#1	#26	#30	#36	#37	#38	DMSO	#1	#26	#30	#36	#37	#38
Time (min)														
T20	21 ± 1	32 ± 3*	32 ± 2*	31 ± 1**	33 ± 4*	42 ± 6**	37 ± 11*	8 ± 2	17 ± 3*	19 ± 3*	18 ± 2*	20 ± 3*	30 ± 4**	29 ± 4**
T40	22 ± 3	32 ± 3	39 ± 6	35 ± 4	35 ± 4	42 ± 7*	37 ± 8*	7 ± 2	18 ± 3*	29 ± 6**	23 ± 3**	23 ± 5*	30 ± 3***	30 ± 4**
T60	20 ± 2	32 ± 3*	36 ± 4*	32 ± 4*	36 ± 4*	46 ± 13*	43 ± 11*	6 ± 1	18 ± 4*	24 ± 5*	21 ± 4*	23 ± 5*	36 ± 11**	39 ± 9**
T90	20 ± 2	33 ± 4	34 ± 4*	35 ± 4*	34 ± 3*	46 ± 12**	43 ± 14*	6 ± 2	19 ± 4*	23 ± 4**	23 ± 5*	20 ± 3**	35 ± 7**	39 ± 11**
T120	20 ± 2	31 ± 4	33 ± 5	30 ± 3	34 ± 4*	40 ± 6**	41 ± 14*	7 ± 1	18 ± 3*	23 ± 6*	17 ± 5	24 ± 4*	29 ± 2***	40 ± 11**
T180	22 ± 2	32 ± 3	35 ± 4	33 ± 3	36 ± 4	41 ± 6**	38 ± 8*	7 ± 2	20 ± 4*	24 ± 4**	21 ± 3**	24 ± 4*	28 ± 1***	33 ± 5***

*n* = 5 (five different samples from five individuals). Role of semen analysis in subfertile couples.

*P < 0.05, **P < 0.01 and ***P < 0.001 with respect to control.

Specifically, for total motility, cells incubated in compounds #37 and #38 were significantly different from the controls at each time point (Table I). Cells incubated in compound #36 were significantly different from the control at T20, T60, T90 and T120. Cells incubated in compounds #26 and #30 were significantly different from the control at T20, T60, T90. Cells incubated in compound #1 were not consistently significantly different over time. Specifically for progressive motility, cells incubated in all compounds were significantly different from the control at each time point (Table I).

#### Removal of compounds

Following incubation for 20 min, the samples were washed (to simulate removal of compound for clinical use prior to, for example, IUI) and incubated for up to 300 min. There was still a significantly higher total and progressively motility compared with controls at the end of incubation with each compound (Table II).

**Table II:** Effects of compounds on sperm motility after compound was removed after 20 min of treatment under non-capacitating conditions.

	Total motility (% ± SEM)							Progressive motility (% ± SEM)						
	DMSO	#1	#26	#30	#36	#37	#38	DMSO	#1	#26	#30	#36	#37	#38
Time (min)														
T20	20 ± 1	35 ± 0***	38 ± 2**	36 ± 2**	37 ± 1***	42 ± 2***	40 ± 2***	8 ± 1	21 ± 1***	24 ± 3**	20 ± 3**	24 ± 1***	27 ± 2***	28 ± 2***
W0	18 ± 1	29 ± 3*	28 ± 3*	26 ± 4	28 ± 3*	30 ± 2**	31 ± 3**	7 ± 1	18 ± 3*	16 ± 3*	14 ± 3	18 ± 3*	19 ± 2**	19 ± 3**
W20	21 ± 3	28 ± 2	33 ± 4*	24 ± 3	31 ± 3*	36 ± 5*	33 ± 5	10 ± 2	17 ± 3	20 ± 4	11 ± 3	20 ± 3*	24 ± 3*	22 ± 5
W40	18 ± 2	29 ± 2**	30 ± 4*	30 ± 4*	27 ± 3*	33 ± 4*	36 ± 2**	7 ± 1	18 ± 3*	19 ± 4*	14 ± 3	17 ± 3*	23 ± 4**	24 ± 2***
W60	18 ± 2	28 ± 4	32 ± 4*	25 ± 3	29 ± 3*	35 ± 2**	32 ± 4*	7 ± 1	16 ± 4	19 ± 3*	13 ± 3	19 ± 3**	23 ± 3**	22 ± 3**
W120	18 ± 2	26 ± 3*	35 ± 4**	25 ± 4	33 ± 6*	34 ± 4**	34 ± 5*	8 ± 2	15 ± 2	22 ± 4*	12 ± 4	21 ± 4*	21 ± 3**	23 ± 5*
W180	17 ± 2	30 ± 3*	32 ± 4*	24 ± 3	29 ± 3*	34 ± 3**	33 ± 4**	7 ± 2	18 ± 3*	21 ± 5*	12 ± 3	20 ± 3**	22 ± 3**	23 ± 3**
W240	18 ± 2	30 ± 2**	31 ± 4*	24 ± 4	29 ± 4*	34 ± 3**	31 ± 4*	9 ± 1	17 ± 3*	20 ± 4*	12 ± 3	21 ± 4*	24 ± 3**	22 ± 4*
W300	15 ± 1	27 ± 2**	30 ± 4*	26 ± 4*	28 ± 4*	33 ± 3**	32 ± 5*	6 ± 2	15 ± 3*	19 ± 4*	13 ± 4	19 ± 4*	21 ± 3**	22 ± 4*

*n* = 4 (four different samples from four individuals). T20 = incubation for 20 min, W0 = compound washed off/removed (min).

*P < 0.05, **P < 0.01 and ***P < 0.001 with respect to control.

Specifically for total motility, cells incubated in compound #1, #26, #36, #37 and #38 showed a significant increase in comparison with the control, at each time point except at W20 (compound #38) and W20 and W60 (compound #1). There was no significant difference in cells incubated in compounds before (T20) and after wash off (i.e. W0-W300). Cells incubated in compound #30 did not show a consistent significant difference over time.

For progressive motility, cells incubated with all compounds showed a significant increase at T20. After washing, cells incubated in compounds #36 and #37 showed a significant increase at each time point. Cells incubated in compound #30, however, do not show any significant difference compared with control. Those incubated in compound #1 were not consistently significantly different over time.

#### Patient samples prepared in NCM

In total, 23 patients were analysed in this category (Table III). The patients were attending for either routine diagnostic andrology assessment or IVF/ICSI and a portion of their semen sample was examined following density gradient centrifugation (as above) and analysed. It was not always possible to compare all six compounds in a single clinical sample due to limitations in the number of sperm available. However, whilst each patient is an individual, there are some general trends. In 3/8 patients with high total motility (WHO normal) there was no response (or a negative response) to the compounds (#1, #26, #30, #37 and #38). There was a noticeable number who showed a significant increase in progressive motility to some, but not all compounds. In contrast, in patients with low progressive motility, there were a higher proportion of patient samples that responded positively compared with those in the normal category. In patient samples with low total motility and low progressive motility, there was a positive response, either by an increase in total motility and/or progressive motility to some, but not all compounds. In this category, only one patient showed no response to any compound (sample ID: 760).

**Table III:** Summary matrix of patient samples (NCM) treated with selected compound(s).

Sample ID	WHO category	#1		#26		#30		#36		#37		#38	
		TM	PM	TM	PM	TM	PM	TM	PM	TM	PM	TM	PM
Decreasing total motility													
721	WHO normal	↓	↓	―	―	―	―	―	―	―	―	―	―
710	WHO normal	―	―	↑	―	↑	―	―	↓	↑	―	↑	―
716	WHO normal	―	↑	―	↑	―	↑	―	↑	―	↑	―	↑
663	WHO normal	―	↑	↑	↑	↑	↑	↑	↑	↑	↑	↑	↑
734	WHO normal	―	―	―	―	―	―			―	―	―	―
736	WHO normal	―	―	―	―	―	―			―	―	―	―
774	WHO normal	―	↑	―	↑	―	↑	―	―	―	↑	―	↑
717	WHO normal	―	―	―	↑	―	↑	―	↑	―	―	―	―
557	Borderline	↑	―	↑	↑	↑	↑	↑	↑	↑	↑	↑	↑
PT01	Borderline			―	↑			↑	↑			―	↑
723	Borderline	―	↑	↑	↑					↑	↑	―	↑
784	Borderline	―	↑	↑	↑	↑	↑	―	↑	↑	↑	―	↑
786	Borderline	↑	↑	↑	↑	↑	↑	―	↑	↑	↑	↑	↑
642	Borderline	↑	↑	↑	↑					↑	↑	↑	↑
653	Borderline	―	↑	↑	↑	↑	↑	↑	↑	↑	↑	↑	↑
769	Borderline	―	―	↑	↑	↑	↑	―	↑	―	↑	―	↑
760	Low TM and PM	―	―	―	―	―	―	―	―	―	―	―	―
782	Low TM and PM			↑	↑					↑	↑	―	↑
708	Low TM and PM	↑	↑	↑	↑	↑	↑	↑	↑	↑	↑	↑	↑
725	Low TM and PM	―	―	↑	―	―	―	―	―	↑	―	↑	―
709	Low TM and PM			↑	↑								
761	Low TM and PM			↑	↑								
729	Low TM and PM			↑	↑								

↑, Significant increase; —, no change; ↓, significant decrease; empty cell, drug was not used in the experiment.WHO normal: WHO normal limits for total motility (40%) and progressive motility (32%), Borderline: borderline motility, low TM and low PM: both total and progressive motility are below the WHO normal limit. No entry means compound is not tested. Significance means SD do not overlap (TM: total motility, PM: progressive motility).

#### IVF patients samples prepared in Quinn's Advantage Fertilization medium

There were 32 patients analysed in this category (Table IV). The patients were attending for IVF and a portion of their density gradient-prepared sample that was used for treatment was obtained for research purposes and analysed. Again, it was not always possible to compare all six compounds due to limitations in the number of sperm available in a clinical sample. As expected, the majority of these patients had normal total and progressive motility (27/32 Table IV). Whilst each patient is an individual, there are some general trends: compounds #37, #38 and #26 were the most effective in increasing the percentage of total motile cells [#26 and #37 (20/32) = 63% and #38 (17/27) = 63% of patients]. As for samples in NCM, the most significant effects were in samples with borderline/low motility.

**Table IV:** Summary matrix of IVF patient samples [Quinn's Advantage^®^ Fertilization (HTF) Medium] treated with selected compound(s).

Sample ID	WHO category	#1		#26		#30		#36		#37		#38	
		TM	PM	TM	PM	TM	PM	TM	PM	TM	PM	TM	PM
Decreasing total motility													
943	WHO normal	―	―	↓	―	↓	―	↓	―	―	―	↓	―
1232	WHO normal			―	―	―	―	―	―	―	↓	―	↓
1308	WHO normal			↑	―	↑	―	―	―	↑	―	―	↓
997	WHO normal	↓	↓	↑	↑	↑	↑			↑	↑	↑	↑
947	WHO normal	―	↑	―	↑	―	↑	―	―	―	―	―	↑
1212	WHO normal			↑	↑	↑	―	↑	―	―	―	↑	―
939	WHO normal	―	↑	↑	↑	―	↑	―	―	↑	↑	―	―
985	WHO normal	―	―	―	―	―	―	―	―	―	―	―	―
1020	WHO normal			↑	―					↑	―		
944	WHO normal	―	―	↑	―	―	―	―	―	↓	―	↑	―
1307	WHO normal			↑	―	↑	↑	↑	―	↑	―	↑	―
872	WHO normal	―	―	―	―	―	―	―	↓	―	↓	―	↓
986	WHO normal	―	―	―	―	―	↑	―	―	―	↑	―	↑
1227	WHO normal			↑	↑	↑	↑	↑	↑	↑	↑	↑	↑
1018	WHO normal			↑	↑					↑	―		
1234	WHO normal			↑	↑	↑	↑	↑	―	↑	↑	↑	↑
1290	WHO normal			↑	―	↑	↑	↑	―	↑	―	↑	―
1298	WHO normal			↑	↑	↑	↑	↑	↑	↑	↑	↑	↑
911	WHO normal	↑	↑	↑	↑	↑	↑	↑	―	↑	―	↑	―
865	WHO normal	―	―	―	―	―	―	―	―	―	―	―	―
1302	WHO normal			↑	↑	―	―	―	―	―	―	↑	↑
867	WHO normal	―	―	―	―	↑	↑	―	―	↑	↑	↑	―
919	WHO normal			↑	―					―	―		
992	WHO normal			―	―	―	―			―	―	―	―
937	WHO normal	↑	↑	↑	↑	↑	↑	―	↑	↑	↑	↑	↑
877	WHO normal	―	―	―	↑	―	↑	―	―	↑	↑	↑	↑
1273	Borderline			↑	↑	↑	↑	↑	↑	↑	↑	↑	↑
1037	Borderline			―	―					↑	―		
1019	WHO normal			↑	―					↑	―		
949	Borderline	―	―	―	↑	―	―	―	―	↑	↑	↑	↑
991	Borderline			↑	↑	―	―			↑	↑	↑	↑
891	Low TM and PM			↑	↑					↑	↑	↑	↑

↑, Significant increase; ― no change; ↓, significant decrease; empty cell, drug was not used in the experiment. WHO normal: WHO normal limits for total motility (40%) and progressive motility (32%), borderline: borderline motility, low TM and low PM: both total and progressive motility are below the WHO normal limit. No entry means compound is not tested. Significance means SD do not overlap (TM: total motility, PM: progressive motility).

#### ICSI patients samples prepared in Quinn's Advantage Medium with HEPES

There were only 16 patients in this category and very small sample volumes were obtained and so the analysis is limited (Table V). However, sperm cells incubated in compound #26 or #37 showed, in all but one case (15/16; 13/14, respectively), an increase in progressive motility. In 14/16 and 11/14 cases, there was an increase in total motility for compounds #26 and #37, respectively. For compound #38, 9/11 cases showed an increase in total or progressive motility.

**Table V:** Summary matrix for ICSI patient samples (Quinn's Advantage^®^ Medium with HEPES) treated with selected compound(s).

Sample ID	WHO category	#1		#26		#30		#36		#37		#38	
		TM	PM	TM	PM	TM	PM	TM	PM	TM	PM	TM	PM
Decreasing total motility													
1160	WHO normal			―	―	―	―	↓	↑				
1163	WHO normal			↑	↑	↑	↑			↑	↑	↑	↑
1213	WHO normal			↑	↑	↑	↑	↑	↑				
1251	WHO normal			↑	↑					↑	↑	↑	↑
1309	WHO normal			↑	↑	↑	↑	↑	↑	↑	↑	↑	↑
873	WHO normal	―	―	↑	↑	―	―	―	―	―	―	―	―
1154	Borderline			↑	↑	―	↑			↑	↑	↑	↑
1236	Borderline			↑	↑	↑	↑	↑	↑	↑	↑	↑	↑
1233	Borderline			↑	↑	↑	↑	↑	↑	↑	↑	↑	↑
1162	Borderline			―	↑	―	↑			―	↑	―	↑
1150	Low TM and PM			↑	↑	―	―			―	↑	↑	―
1183	Low TM and PM			↑	↑	↑	↑			↑	↑		
1038	Low TM and PM			↑	↑					↑	↑		
1261	Low TM and PM			↑	↑					↑	↑		
1301	Low TM and PM			↑	↑					↑	↑	↑	↑
1257	Low TM and PM			↑	↑	↑	↑			↑	↑	↑	↑

↑, Significant increase; ― no change; ↓, significant decrease; empty cell, drug was not used in the experiment.

WHO normal: WHO normal limits for total motility (40%) and progressive motility (32%), borderline: borderline motility, low TM and low PM: both total and progressive motility are below the WHO normal limit. No entry means compound is not tested. Significance means SD do not overlap (TM: total motility, PM: progressive motility).

## Discussion

Using a two-stage comprehensive approach, we have successfully identified several compounds that have robust and effective stimulation of sperm motility, are non-toxic, initiate a functional improvement as judged by Kremer testing and importantly have a positive response on a significant proportion of patient samples prepared under clinical conditions.

This study used a two-phase strategy. In Phase 1, 43 commercially available compounds with reported PDEI activity were screened for their effects on sperm motility using CASA. Pooled samples from three to four different donors were utilized to reduce variability and increase the number of cells available for simultaneous examination of multiple compounds (usually three to five in each run). Cells in the 40% fraction (those with poor motility) were used as putative surrogates for patient samples. Previous studies have suggested that these cells have a similar profile, in terms of motility, morphology and DNA status, to men with sperm dysfunction/male infertility (O'Connell *et al.*, 2003; Glenn *et al.*, 2007). The first screening was performed with NCM, as these are the conditions normally used for IUI (Bjorndahl *et al.*, 2010). Moreover, an incubation time of 20 min was designed to fit with clinical procedures for sperm preparation. In general, consistent results on sperm motility were obtained. In Phase 1, the effects of the six leading compounds were determined using pooled samples. Experiments on individual samples then showed a similar profile of results to pooled samples, and, notably showed a significant increase in both total and progressive motility (Fig. 3A and B; Supplementary data, Fig. S1). Additionally, consistent stimulation of total and progressive motility were obtained when the cells were incubated under capacitating conditions (Fig. 3C).

The objective of Phase 1 was to allow a large number of compounds to be screened relatively efficiently in order to identify potential hit targets for further study. Phase 2 consisted of a more detailed assessment based around guidelines for the testing of compounds that can potentially be considered safe for clinical use (Mortimer *et al.*, 2013). Phase 2 involved sperm function testing with a view to the use of the compounds in ART, e.g. IUI. Modified Kremer testing demonstrated that the stimulation in motility was also of functional benefit, i.e. higher numbers of cells penetrated the viscous media. Importantly, the compounds did not appear to have a significant negative effect as there was no significant induction of the AR (Supplementary data, Fig. S2) or PS exposure (Supplementary data, Fig. S2). This is consistent with the finding of motility maintenance over a significant time course, even with continuous incubation (Table I). Whilst the six key compounds selected for Phase 2 had positive effects, there were differences in the efficacy suggesting fewer lead candidates for future clinical use. For example, compounds #26, #37 and #38 had the most significant effect on Kremer testing which is broadly consistent, at least for compounds #37 and #38 with the positive effect on motility over time (Table I). Clinical use of the compounds would involve washing and effective removal prior to use. Table II demonstrates that stimulation of total motility was maintained over time; however, progressive motility was not consistently affected using compounds #1 and #30. Continual incubation (Table I) suggests that the positive effect on total motility of compounds #1 and #26 and #30 were not maintained throught incubation. For progressive motility, particular stimulation with compounds #38 and #37 was observed (Table I).

The fundamental clinical aim is to translate what happens in an experimental model to effects in patient samples. To address this, we tested a spectrum of diagnostic and treatment samples under both non-capacitating and capacitating conditions (Tables III–V). In general, in samples with good motility, e.g. IVF, there was a minimal effect on total motility but, in some cases, a noteable effect on progressive motility. In contrast, in samples with lower motility, there was a significant effect on both total and progressive motility. These clinical data give some indications as to the possible therapeutic use and effectiveness. Generally, there appears to be limited benefit for samples with good motility, as expected and consistent with previous data using PTX (Nassar *et al.*, 1999). In cells incubated under non-capacitating conditions 15/23 and 17/23 of the samples responded to compound #26 with regard to total and progressive motility. From the limited data available, compounds #1 and #30 were less effective. In samples incubated under capacitating conditions (Table IV), compounds #37, #38 and #26 were the most effective in increasing the percentage total motile cells (∼63% of samples) and, as for samples in NCM, the most significant effects were in samples with borderline/low motility. Only relatively few ICSI samples were examined (Table V); however, in the overwhelming majority of cases, cells incubated in compounds #26, #37 and #38 showed an increase in progressive motility and total motility.

For practical purposes, three concentrations were adopted in Phase 1 using doses of 1, 10 and 100 µM. The objective was to determine which concentration was the most effective under these conditions (non-capacitating conditions with cells in the 40% fraction). Concentrations of 1 and 10 µM did have pro-motility effects, in some cases, but it was very much less (data not shown) and as such Phase 2 only used PDEIs at a concentration of 100 µM. For some of the compounds tested, 100 µM is much greater than the reported IC_50_ (e.g compound #1), whilst for others (e.g. compound #26) it is comparable (see Supplementary data, Table SII). What is interesting is that the reported IC_50_ for a number of compounds varies remarkably perhaps because some are generated using purified recombinant enzymes and others on a wide variety of different cell types (Supplementary data, Table SII). For spermatozoa there are no available data; there is no information on what concentration of compound enters the sperm cytoplasm, whether there are pumps effectively making high external concentrations necessary, the specificity of the sperm PDE complex(s) or which complexes are present (see below). Preliminary experiments on the three most promosing candidates (compounds #26, #37 and #38) utilized concentrations from 0.5 to 100 µM to examine a potential concentration effect on motility and kinematic parameters. For compound #26, progressive motility was significantly stimulated at 20–100 µM (in keeping with the IC_50_ for other cells), whereas progressive motility was signfiicantly stimulated at 1–100 µM for compound #37 and at 0.5–100 µM for compound #38, both of which are within the ranges of the IC_50_ for other cell types (see above and Supplementary data, Figs S3–S5, respectively).

Surprisingly, in view of the plethora of information available on PDEs in other cells, there is a remarkable paucity of studies on the identity, location and nature of PDEs in the human spermatozoon (Conti and Beavo, 2007; Houslay, 2009). The measurement of sperm PDE activity in the presence of inhibitors for PDE-1 (8-MeO-IBMX) (Fisch *et al.*, 1998), PDE-4 (RS 25344) (Fisch *et al.*, 1998), PDE-3 (milrinone) (Lefièvre *et al.*, 2002), PDE-5 (sildenafil) and stimulators for PDE-1 (calcium/CaM) (Lefièvre *et al.*, 2000, 2002) suggests that these PDEs are present in human spermatozoa, although PDE-5 is present at very low levels. mRNA transcripts of PDEs have been detected (Richter *et al.*, 1999) but very few studies examine localization (Lefièvre *et al.*, 2002) and there are minimal data on protein expression. In fact, proteomic studies of human sperm reveal a paucity of PDE in spermatozoa (Baker *et al.*, 2013; Wang *et al.*, 2013). There are no studies examining the role of defective PDEs in sperm dysfunction, e.g. aberrant expression. In view of the high concentrations of compounds used in this study the specificity of effect on PDEs is also uncertain. Other biochemical pathways could be affected and as such we do not know if the biological effect is via PDE and/or another mechanism. Notwithstanding the clinical end-point is real: there are significant changes in movement without an adverse effect on sperm function; however, more detailed biochemical studies are required to ascertain the mechanism(s) of action.

In view of the above it is perhaps not surprising that of the six key compounds identified as potential clincial candidates (dipyridamole, ibudilast, 8-MeO-IBMX, etazolate hydrochloride, papaverine and tofisopam) there is a noteable lack of data on human sperm. No information is available on dipyridamole, ibudilast or tofisopam. Etazolate hydrochloride, which is reported as a selective PDE-4 inhibitor (as SQ20009), increases cAMP in hamster sperm (Mrsny *et al.*, 1984) and phosphorylation of membrane proteins (presumably as part of capacitation) in humans (Huacuja *et al.*, 1977), although there are no data on motility. 8-MeO-IBMX, reported as a specific inhibitor of calmodulin-sensitive cyclic GMP PDE, has been used in mice fertilization studies (Baxendale and Fraser, 2005) but there are no reports of effects on the motility of human spermatozoa. Papaverine, reported as a PDE-10A inhibitor, has been used at a concentration of 500 µM to increase cyclic nucleotides and mimic the effects of capacitation in human sperm. After 5 min of incubation, there was an increased calcium response to progesterone (Torres-Flores *et al.*, 2008). Papaverine has also been used to mimic capacitation changes by modulating the cAMP pathway in boars (Harrison, 2004) and guinea pig sperm (Hunnicutt *et al.*, 2008).

In conclusion, we have successfully identified several compounds that have robust and effective stimulation of sperm motility, are non-toxic to the cells, initiate a functional improvement as judged by Kremer testing and importantly have a positive response on a significant proportion of patient samples under clinical conditions of treatment. Ibudilast, papaverine and tofisopam appear to be very promising candidates but further experiments are still necessary to establish safety and clinical effectiveness, e.g. IVF and/IUI trials. There are significant challenges with screening for the effects of a large number of compounds on human spermatozoa. CASA is not well suited to traditionally high-throughput screening. In the long term, if significant progress is to be made in understanding sperm function, there is a genuine need to develop a high-throughput assay which would enable the rapid screening of thousands of compounds.

## Supplementary data

Supplementary data are available at http://humrep.oxfordjournals.org/.

## Authors' roles

S.T. screened all 43 compounds in Phase I and 6 in Phase II using functional testing and designed the AR and annexin V experiments. O.A.M. performed additional sperm function testing, examined a number of patient samples and was involved in the initial analysis of the data and experimental design. L.F. performed a number of capacitation/non-capacitation experiments on donors in Phase 1. P.G.W. and C.L.R.B. designed the study and obtained funding for the experiments. L.L. was involved in the initial concept of the study and provided advice regarding assessments, analysis and experimental modification and analysis. S.J.M.D.S. was involved in the recruitment and consent of patients, the experimental design, data analysis, patient selection and clinical significance. S.G.B. contributed to writing of the manuscript. All authors contributed to the editing of the final manuscript. All authors approved the final manuscript for submission.

## Funding

Work in the authors' laboratories is funded by MRC, The Wellcome Trust, TENOVUS (Scotland), University of Dundee, NHS Tayside and Scottish Enterprise. Resources from a MRC (Developmental Pathway Funding Scheme–Principal investigators C.L.R.B. and P.G.W.) ‘Enhancement of human sperm function (motility) using modulators of cAMP and cGMP [phosphodiesterase–PDE Inhibitors (PDEI's)] primarily funded the data presented in this study. Sarah J Martins Da Silva is a NHS Scotland Research Fellow. Lorna Frame was funded by Jean Shanks Foundation.

## Conflict of interest

An UK Patent application containing part of the described work was obtained on 10th October 2011 as a PCT/GB2012/052514 (patent application number GB1117453.9) ‘Improved Sperm Function/activity’—inventors Christopher LR Barratt and Paul G Wyatt. This was published as patent #WO2013054111 A1 in 2013. Steve Tardif, Oladipo A Madamidola, Sean G Brown, Lorna Frame, Linda Lefièvre and Sarah J Martins Da Silva have no conflicts of interests to declare.

## Supplementary Material

Supplementary Data
