# Supplementary material for: Clinically relevant enhancement of human sperm motility using compounds with reported phosphodiesterase inhibitor activity
Source: Hum Reprod. 2014 Aug 14;29(10):2123–35. doi: 10.1093/humrep/deu196 (PMC4481575; doi:10.1093/humrep/deu196)
Supplement: Supplementary Data [file supp_29_10_2123__index.html]

Clinically relevant enhancement of human sperm motility using compounds with reported phosphodiesterase inhibitor activity — Clinically relevant enhancement of human sperm motility using compounds with reported phosphodiesterase inhibitor activity — Supplementary Data 

# Clinically relevant enhancement of human sperm motility using compounds with reported phosphodiesterase inhibitor activity

## Supplementary Data

Supplementary Data

**Files in this Data Supplement:**

- Supplementary Figure 1 - pdf file
- Supplementary Figure 2 - pdf file
- Supplementary Figure 3 - pdf file
- Supplementary Table 1 - pdf file
- Supplementary Table 2 - pdf file
