# Supplementary material for: Clinically relevant enhancement of human sperm motility using compounds with reported phosphodiesterase inhibitor activity
Source: Hum Reprod. 2014 Aug 14;29(10):2123–35. doi: 10.1093/humrep/deu196 (PMC4481575; doi:10.1093/humrep/deu196)
Supplement: Supplementary Data [file supp_deu196_deu196supp_fig1.pdf]

Human Reproduction, Vol.0, No.0 pp. 1–8, 2014
doi:10.1093/humrep/deu196

human reproduction

SUPPLEMENTARY DATA

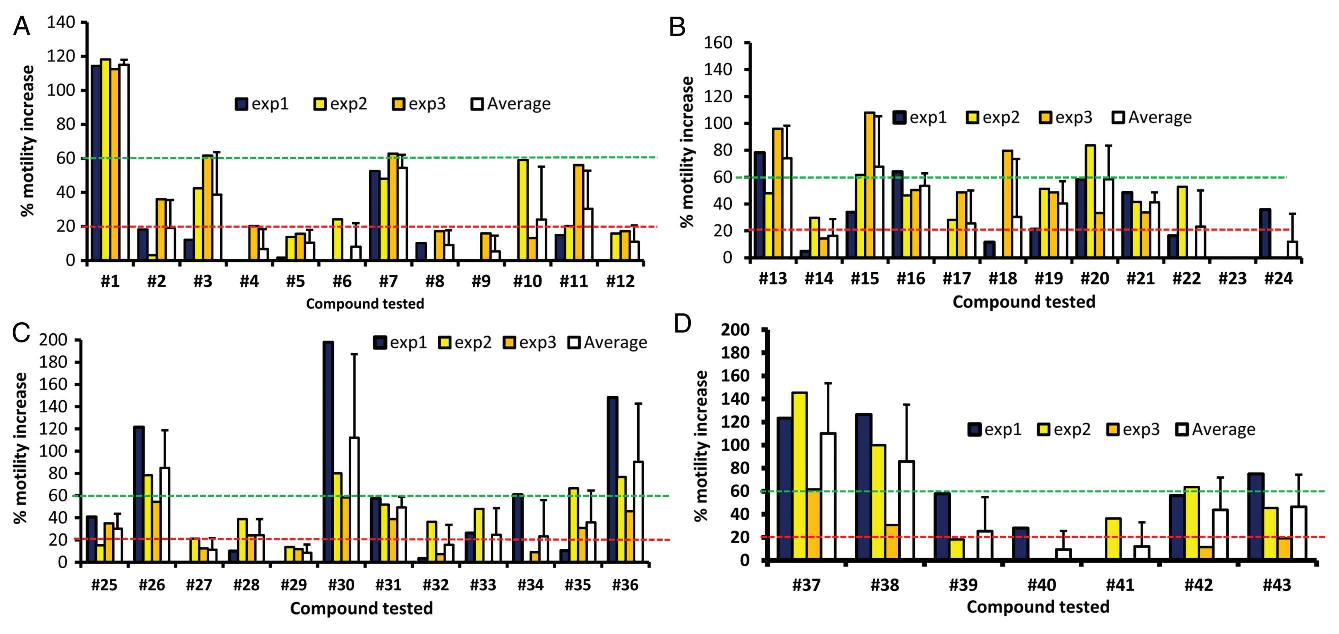

Supplementary Figure S1 Effect(s) of compound(s) on sperm motility: spermatozoa were treated for 20 min at 37°C with 100 μM of compound. (**A**) Compound tested (#1–#12). (**B**) Compound tested (#13–#24). (**C**) Compound tested (#25–#36). (**D**) Compound tested (#37–#43). A 100% increase equals a 2-fold increase in motility compared with 1% DMSO negative control (i.e. if DMSO = 15%, treatment = 30%); $n = 3$ (three separate analysis of pooled sample), mean ± SD. The green line indicates threshold for strong responder, while the red line is the threshold for background. Compounds between the green and red lines indicate mild responder.
