# Supplementary material for: Clinically relevant enhancement of human sperm motility using compounds with reported phosphodiesterase inhibitor activity
Source: Hum Reprod. 2014 Aug 14;29(10):2123–35. doi: 10.1093/humrep/deu196 (PMC4481575; doi:10.1093/humrep/deu196)
Supplement: Supplementary Data [file supp_deu196_deu196supp_fig2.pdf]

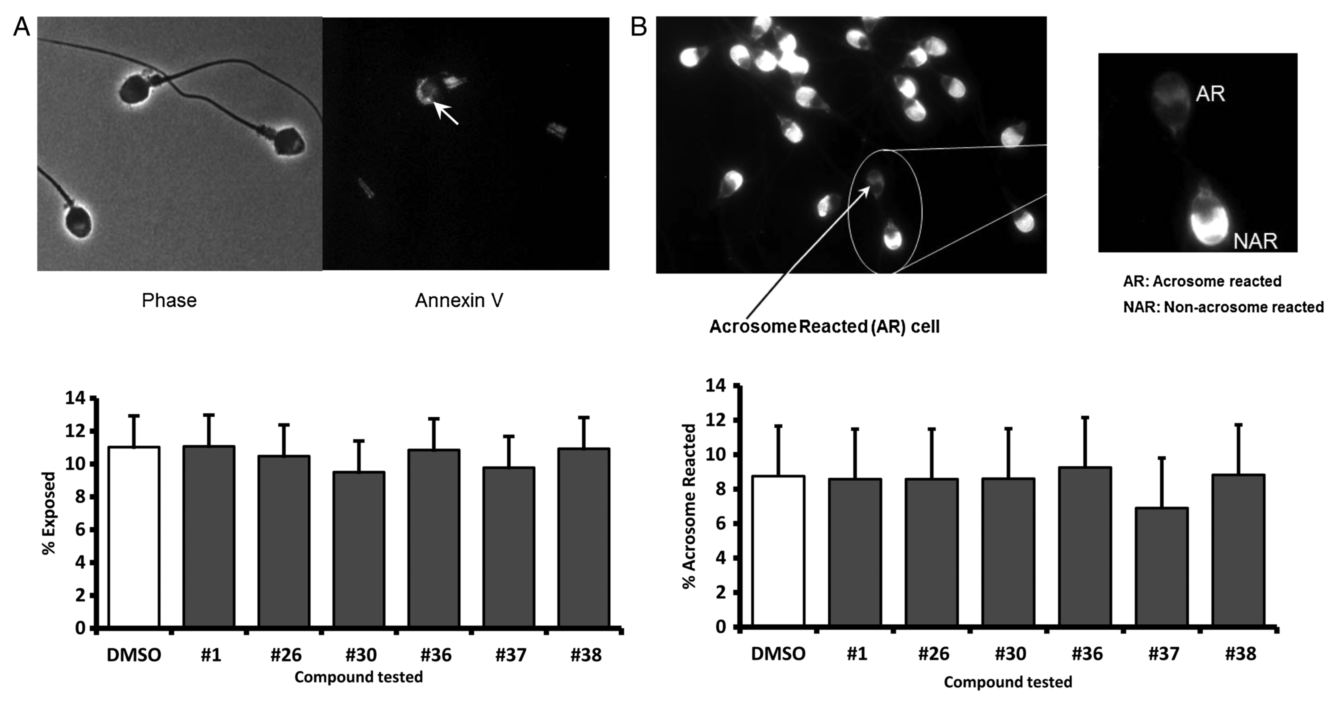

Supplementary Figure S2 Effect(s) of selected compound on the expression of phosphatidylserine (PS) and percentage of acrosome-reacted spermatozoa: spermatozoa (40% fraction: poor motility population) were treated for 20 min at 37°C with 100 μM of compound. (**A**) Sperm cells were labelled with fluorescein isothiocyanate-labelled annexin V/propidium iodide (PI). The arrow shows cell with no externalization of phosphatidylserine ($n = 4$, four samples from four individuals; $P = 0.6$. mean ± SEM). (**B**) Permeabilized spermatozoa were labelled with *Pisum sativum lectin* ($n = 4$ four samples from four individuals; $P = 0.6$ mean ± SEM). Images represent typical image used for criteria for scoring. Minimum of 200 cells counted. Magnification ×400.
