# Supplementary material for: Clinically relevant enhancement of human sperm motility using compounds with reported phosphodiesterase inhibitor activity
Source: Hum Reprod. 2014 Aug 14;29(10):2123–35. doi: 10.1093/humrep/deu196 (PMC4481575; doi:10.1093/humrep/deu196)
Supplement: Supplementary Data [file supp_deu196_deu196supp_fig3.pdf]

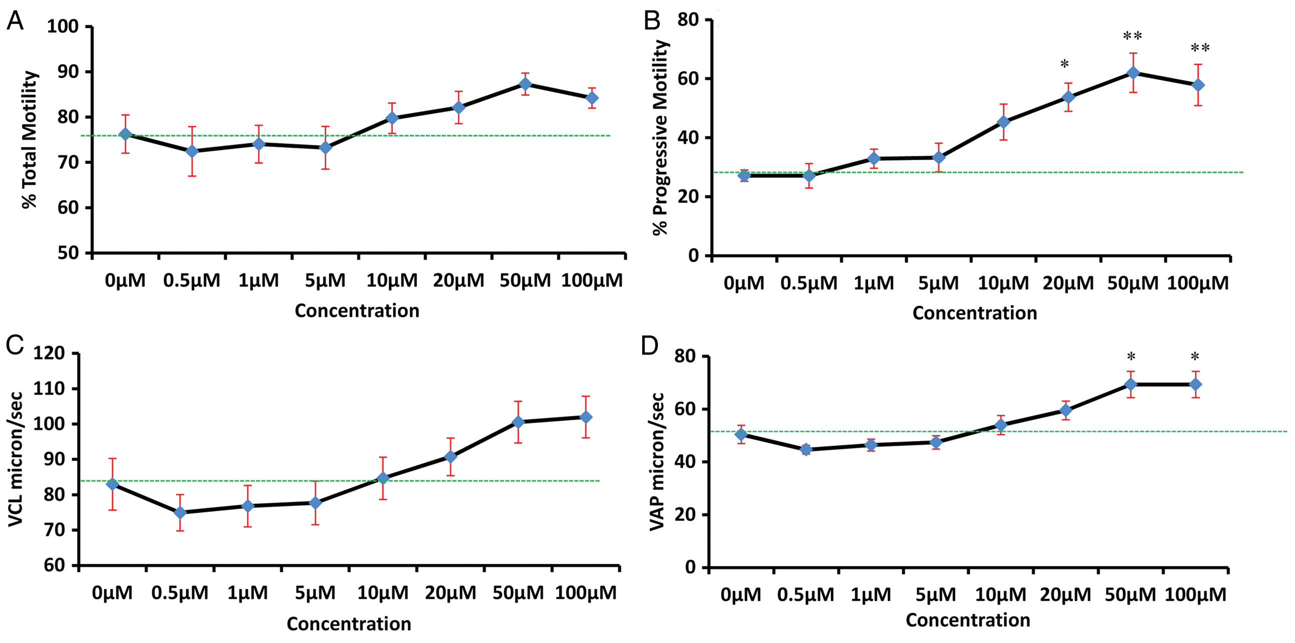

Supplementary Figure S3 Determination of minimum effective concentration of compound #26 on human sperm: The population of highly motile human spermatozoa was isolated by direct swim-up. Cells were incubated in NCM at 37°C for 1 h; they were then treated with compound #26 (final concentrations of 100, 50, 20, 10, 5, 1 and 0.5 µM) or without compound (NCM, 0 µM: non-capacitating media) and left to incubate for 20 min at 37°C (**A**) Percentage total motility. (**B**) Percentage progressive motility. (**C**) Curvilinear velocity. (**D**) Average path velocity $n = 4$ (four different samples from two individuals), mean ± SEM; one-way ANOVA (Tukey's honest significant difference), $*P < 0.05$ and $**P < 0.01$. The green line indicates 'cut off' represented by average value of control sample (NCM).

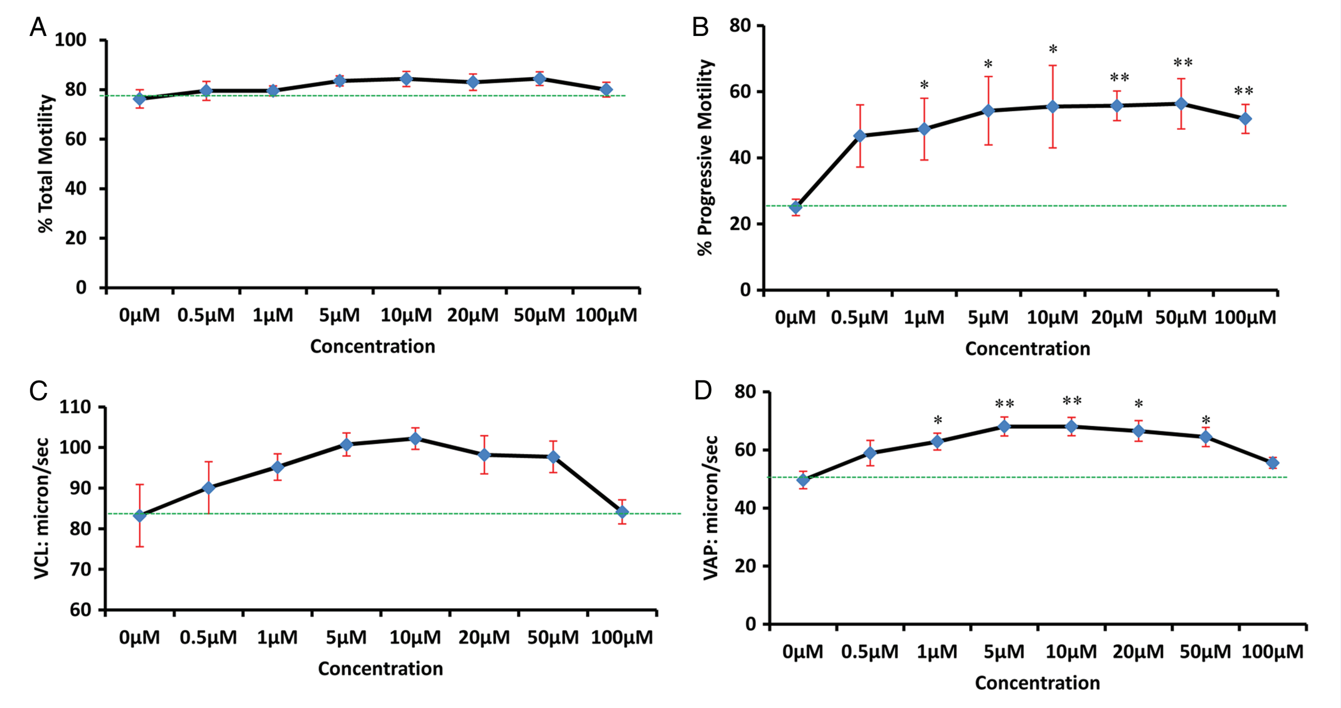

Supplementary Figure S4 Determination of minimum effective concentration of compound #37 on human sperm: The population of highly motile human spermatozoa was isolated by direct swim-up. Cells were incubated at 37°C for 1 h; they were then treated with compound #37 (final concentrations of 100, 50, 20, 10, 5, 1 and 0.5 µM) or without compound (NCM, 0 µM: non-capacitating media) and left to incubate for 20 min at 37°C (**A**) Percentage total motility. (**B**) Percentage progressive motility. (**C**) Curvilinear velocity. (**D**) Average path velocity, $n = 4$ (four different samples from two individuals), mean ± SEM; one-way ANOVA (Tukey's honest significant difference), $*P < 0.05$ and $**P < 0.01$. The green line indicates 'cut off' represented by average value of control sample (NCM).

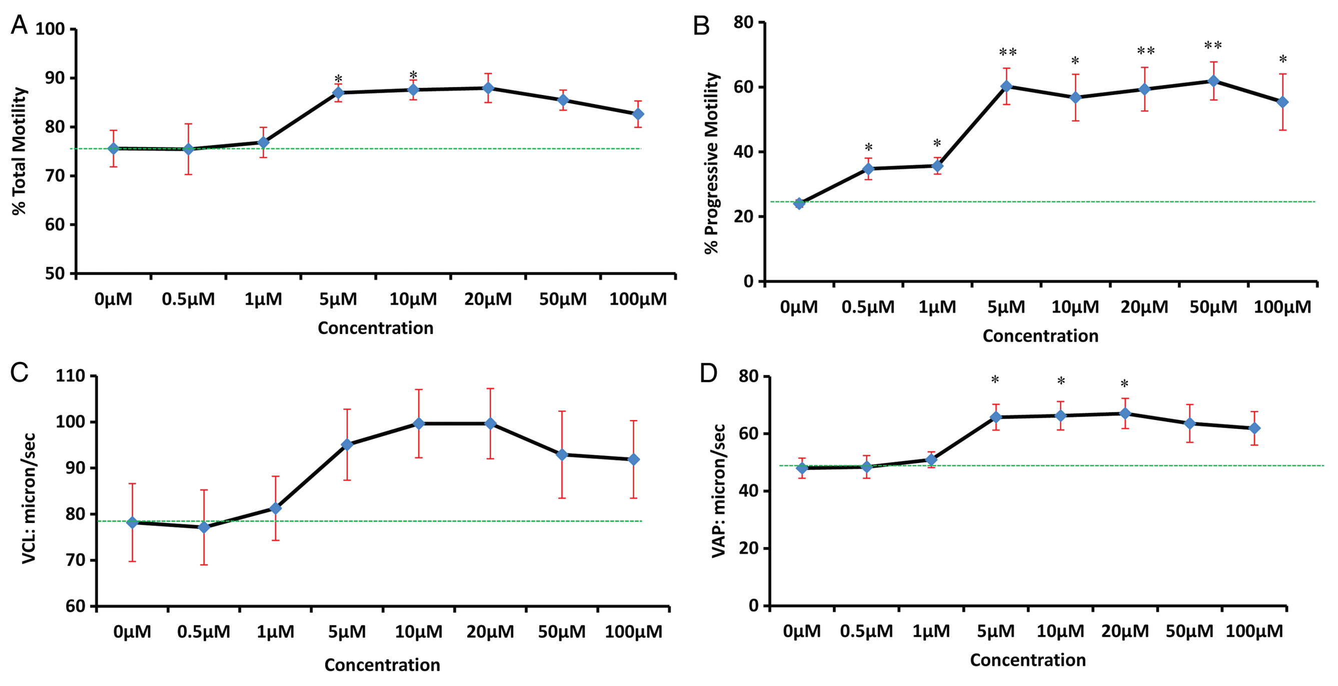

Supplementary Figure S5 Determination of minimum effective concentration of compound #38 on human sperm: The population of highly motile human spermatozoa was isolated by direct swim-up. Cells were incubated at 37°C for 1 h; they were then treated with compound #38 (final concentrations of 100, 50, 20, 10, 5, 1 and 0.5 μM) or without compound (NCM, 0 μM: non-capacitating media) and left to incubate for 20 min at 37°C (**A**) Percentage total motility. (**B**) Percentage progressive motility. (**C**) Curvilinear velocity. (**D**) Average path velocity, $n = 3$ (three different sample from two individuals), mean ± SEM; one-way ANOVA (Tukey's honest significant difference), $*P < 0.05$ and $**P < 0.01$. The green line indicates 'cut off' represented by average value of control sample (NCM).
