# Supplementary material for: Clinically relevant enhancement of human sperm motility using compounds with reported phosphodiesterase inhibitor activity
Source: Hum Reprod. 2014 Aug 14;29(10):2123–35. doi: 10.1093/humrep/deu196 (PMC4481575; doi:10.1093/humrep/deu196)
Supplement: Supplementary Data [file supp_deu196_deu196supp_table1.pdf]

Supplementary Table S1 List of compounds screened during Phase 1.

Tag #	Product name	Tag #	Product name
1	Dipyridamole	23	A-7 hydrochloride
2	(R)-(-)-Rolipram	24	Zaprinast
3	Rolipram	25	Cilostazol
4	Vinpocetine	26	Ibudilast
5	CGH 2466 dihydrochloride	27	Irsogladine maleate
6	W-7 hydrochloride	28	Caffeine
7	IBMX	29	CP 80633
8	Milrinone	30	MMPX (8-MeO-IBMX)
9	BRL 50481	31	Siguazodan
10	Mesopram	32	EHNA hydrochloride
11	Cilostamide	33	Theophylline
12	Pentoxifylline	34	YM 976
13	T 0156 hydrochloride	35	ICI 63197
14	(S)-(+)-Rolipram	36	Etazolate hydrochloride
15	Zardaverine	37	Papaverine
16	RS 25344 hydrochloride	38	Tofisopam
17	Anagrelide hydrochloride	39	Nicardipine
18	MY-5445	40	Nimodipine
19	Ro 20-1724	41	Tadalafil
20	Sildenafil citrate	42	Bay-73-6691
21	Trequinsin hydrochloride	43	Bay-60-7550
22	W-9 hydrochloride		
