# Supplementary material for: Clinically relevant enhancement of human sperm motility using compounds with reported phosphodiesterase inhibitor activity
Source: Hum Reprod. 2014 Aug 14;29(10):2123–35. doi: 10.1093/humrep/deu196 (PMC4481575; doi:10.1093/humrep/deu196)
Supplement: Supplementary Data [file supp_deu196_deu196supp_table2.pdf]

Supplementary Table SII **Compound structure and established IC_{50} of lead compound(s).**

Compound	Structure	PubChem compounds
#1 Dipyridamole	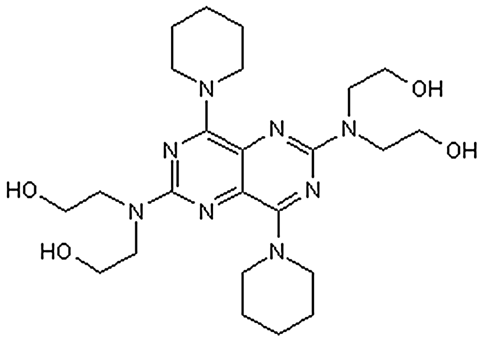	Chemical structure ID: 3108 Substance summary ID: 855977 103242914 BioActivity Types: IC_{50} [μM] BioAssay Name: [IC_{50}: 21.0682 AID: 712], [IC_{50}: 0.015 AID 592230 PubMed ID:21366300], [IC_{50}: 0.083 AID 625197], [IC_{50}: 0.1448 AID 296876 PubMed ID:17636949], [IC_{50}: 0.34 AID 247741 PubMed ID:15369395], [IC_{50}: 0.382 AID 625261], [IC_{50}: 0.5 AID 159630 PubMed ID:9873613], [IC_{50}: 0.52 AID 220176 PubMed ID:8254606], [IC_{50}: 0.574 AID:158100 PubMed ID:10891111], [IC_{50}: 1.579 AID:625166] [IC_{50}: 2.831AID 625167], [IC_{50}: 3.24 AID:159068 PubMed ID:10891111], [IC_{50}: 3.6 AID:158595 PubMed ID:8254606], [IC_{50}: 4 AID:158290 PubMed ID:2985781], [IC_{50}: 5.27 AID:159948 PubMed ID:10891111], [IC_{50}: 6.4 AID:158586 PubMed ID:8254606], [IC_{50}: 6.956 AID:625146], [IC_{50}: 43 AID:158302 PubMed ID:2985781], [IC_{50}: 44 AID:159366 PubMed ID:10891111], [IC_{50}: 45 AID:158887 PubMed ID:2985781]
#26 Ibudilast	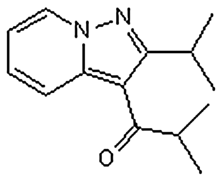	Chemical structure ID: 3671 Substance summary ID: 103185681 BioActivity Types: IC_{50} [μM] BioAssay Name: [IC_{50}: 0.054 AID:363371 PubMed ID:18686943], [IC_{50}: 0.065 AID:363372 PubMed ID:18686943], [IC_{50}: 0.166 AID:363151 PubMed ID:18686943], [IC_{50}: 0.239 AID:363373 PubMed ID:18686943], [IC_{50}: 5 AID:598348 PubMed ID:21530250]
#30 MMPX	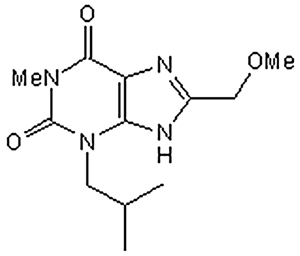	Chemical structure ID: 155806 Substance summary ID: 103267103 BioActivity Types: IC_{50} [μM] BioAssay Name: [IC_{50}: 0.166 AID:158581 PubMed ID:2423691], [IC_{50}: 0.166 AID:158720 PubMed ID:6276544], [IC_{50}: 0.166 AID:158752 PubMed ID:9216839], [IC_{50}: 0.166 AID:157927 PubMed ID:9216839], [IC_{50}: 0.166AID:349550 PubMed ID:19303290]
#36 Etazolate hydrochloride	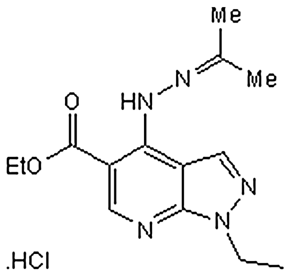	Chemical structure ID: 37274 Substance summary ID: 135727535 BioActivity Types: IC_{50} [μM] BioAssay Name: No information on IC_{50}
#37 Papaverine hydrochloride	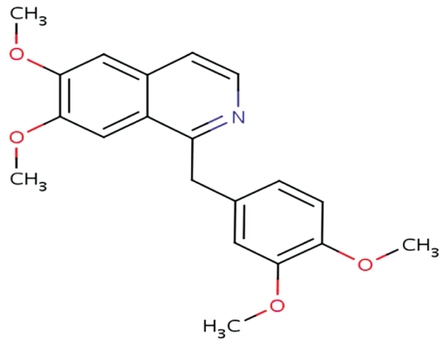	Chemical structure ID: 4680 Substance summary ID: 103183767 BioActivity Types: IC_{50} [μM] BioAssay Name: [IC_{50}: 0.017 AID:550507 PubMed ID:21087867]*,[IC_{50}: 0.021 AID:615719, IC_{50}: 0.036 AID:615724 PubMed ID:21705115]*, [IC_{50}: 0.036 AID:650201 PubMed ID:22321214]*, [IC_{50}: 0.21 AID:598564 PubMed ID:21602043]*, [IC_{50}: 0.55 AID:219692 PubMed ID:6090662], [IC_{50}: 0.62 AID:158594 PubMed ID:8254606], [IC_{50}: 0.665 AID:625165], [IC_{50}: 0.762 AID:625247], [IC_{50}: 0.817 AID:625166], [IC_{50}: 0.917 AID:615720 PubMed ID:21705115], [IC_{50}: 1.03 AID:615721 PubMed ID:21705115], [IC_{50}: 1.1 AID:628752 PubMed ID:21920746], [IC_{50}: 1.55 AID:269391 PubMed ID:16784847], [IC_{50}: 1.55 AID:380881 PubMed ID:10479332], [IC_{50}: 1.7 AID:158589 PubMed ID:8254606], [IC_{50}: 2.3 AID:158598 PubMed ID:8254606], [IC_{50}: 4.23 AID:399545 PubMed ID:14987052], [IC_{50}: 6.6 AID:158305 PubMed ID:2985781], [IC_{50}: 8.8 AID:220176 PubMed ID:8254606], [IC_{50}: 9 AID:158892 PubMed ID:2985781], [IC_{50}: 12 AID:55210 PubMed ID:6298424], [IC_{50}: 17 AID:158293 PubMed ID:2985781], [IC_{50}: 21 AID:55334 PubMed ID:6298424], [IC_{50}: 21.6 AID:220175 PubMed ID:8254606], [IC_{50}: 24.33 AID:263610 PubMed ID:16483771]

Continued

Supplementary TableSII *Continued*

Compound	Structure	PubChem compounds
#38 Tofisopam	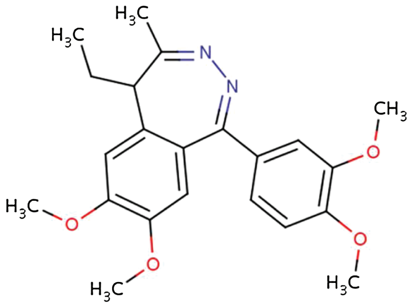	Chemical structure ID: 5502 Substance summary ID: 103555967<br>BioActivity Types: IC_{50} [μM]<br>BioAssay Name:[IC_{50}: 0.68 AID:628752, IC_{50}: 0.9 AID 628754, IC_{50}: 5.9 AID 628755, PubMed ID:21920746]

Chemical structure of selected compounds as identified through PubChem compounds and structure. AID (PubChem's BioAssay identifier) is the Bioassay identifier number that logs the technique and tissue used for determining the IC_{50} of compounds. PubMed ID is the reference related to BioAssay.

*Publication that showed that papaverine hydrochloride targets PDE 10.
